# Gallbladder Agenesis Mimicking Chronic Cholecystitis in a Young Woman

**DOI:** 10.7759/cureus.18222

**Published:** 2021-09-23

**Authors:** Jeffrey S Joseph, Vinith Ramesh, Kholoud K Allaham, Gurubharath Ilangovan, Moien AB Khan

**Affiliations:** 1 Radiology, Chettinad Hospital and Research Institute, Chennai, IND; 2 Family Medicine, College of Medicine and Health Sciences, Al Ain, ARE; 3 Family Medicine, College of Medicine and Health Sciences, United Arab Emirates University, Al Ain, ARE; 4 Primary Care, North West London - National Health Service Provider, London, GBR

**Keywords:** magnetic resonance cholangiopancreatography, biliary colic, gallbladder agenesis, gall blader disease, chronic abdominal pain, abdominal laparoscope, abdominal radiology, direct primary care

## Abstract

Gallbladder agenesis is a rare anatomic congenital abnormality caused by the cystic bud failing to develop into the gallbladder. Gallbladder agenesis has a variable presentation, with 50% of patients presenting with symptoms mimicking biliary colic and 35% being incidentally discovered during surgery or autopsy, while another 15% can present with fatal fetal anomalies.

In this article, we present a case of gallbladder agenesis in a young woman who presented with biliary-colic-like symptoms suggesting cholecystitis. The gallbladder was not well visualized on ultrasonography, simulating chronic cholecystitis due to shrunken or contracted bladder. Further imaging with computed tomography (CT) and magnetic resonance cholangiopancreatography (MRCP) helped in the successful diagnosis of gallbladder agenesis and helped prevent unnecessary surgical intervention.

Due to the lack of clinical suspicion diagnosing gallbladder agenesis preoperatively is still rare. Persistent symptoms are often associated with biliary colic pain leading to surgery. Conservative management consists of using antispasmodic medications. MRCP may be required to rule out gallbladder agenesis and avoid unnecessary surgery.

Gallbladder agenesis can present with symptoms similar to cholecystitis. If the gallbladder is not visualized well on the ultrasound, an additional radiological examination is required. Clinicians’ understanding of the condition helps to accurately diagnose the condition preoperatively using the appropriate investigations, thereby minimizing the operative risk to the patient.

## Introduction

Gallbladder agenesis is a congenital anomaly in the biliary system that was first reported in 1701 by Lemery et al [1]. It is a rare congenital abnormality of the biliary tree with an incidence of <0.1% (range: 0.04%-0.1%) [2] and a female preponderance of 3:1 [1,3]. Literature evidence suggests that only 500 similar clinical cases have been reported [4]. During autopsies, gallbladder agenesis is frequently encountered equally among both males and females, but women are twice as likely to be clinically presenting with symptoms [5].

A number of other congenital anomalies with multisystem involvement can accompany gallbladder agenesis. When associated with other anomalies, the condition can be detected early in childhood. Gallbladder agenesis most often presents as an isolated, non-syndromic entity and is diagnosed often later in life. Furthermore, gallbladder agenesis is associated with, and found in one out of every six cases of, extrahepatic biliary atresia [5].

The rarity of the condition and its uncertain imaging features often leads to misdiagnosis and unwarranted surgical procedures [5]. We present a clinical case of gallbladder agenesis mimicking and presenting as chronic cholecystitis.

## Case presentation

A 20-year-old female was referred to the radiology department with suspicion of cholelithiasis. She had recurrent dyspeptic symptoms with upper abdominal discomfort and bloating. She had three similar episodes of pain involving the upper-right abdominal quadrant and was treated with smooth muscle relaxants and NSAIDs. She had no prior surgical history. There was no relevant family history. No congenital anomalies or syndromes were reported. She was not on any regular medications. The patient was mildly obese, with a BMI of 28 kg/m^2^. On physical examination, there were no signs of jaundice or pallor. Mild tenderness with no guarding was noted upon abdominal palpation in the right hypochondrium. Laboratory investigations, including the liver function tests, were normal.

**Figure 1:**
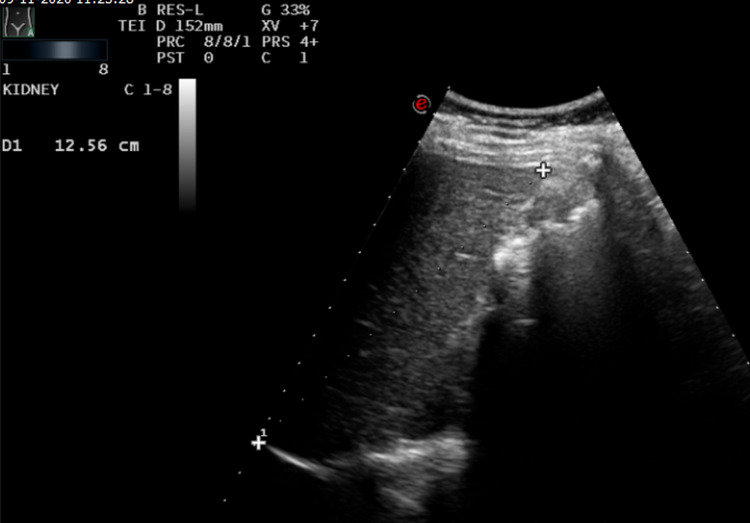
Ultrasound image of the gallbladder fossa showing linear echogenicity representing bowel shadows.

Ultrasound of the abdomen showed normal echotexture of the abdominal solid organs. However, the gall bladder was non-visualized in the gallbladder fossa (Figure 1). There was no evidence of peritoneal free fluid.

**Figure 2:**
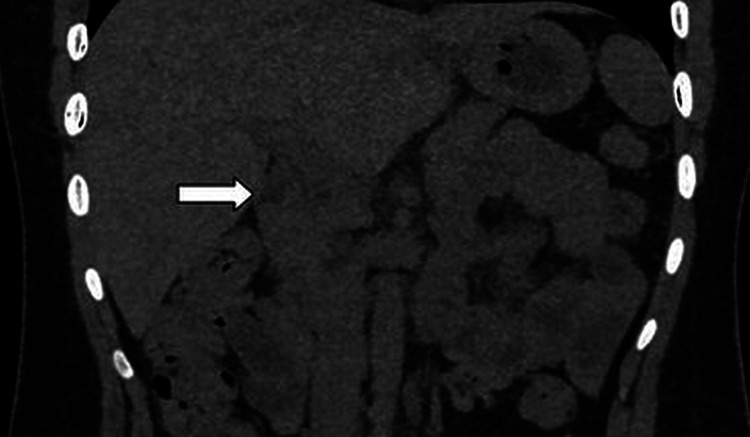
Coronal reformatted non-contrast CT image showing empty gallbladder fossa (arrow). No radio-dense calculi were seen in the biliary system. CT- Computed Tomography

**Figure 3:**
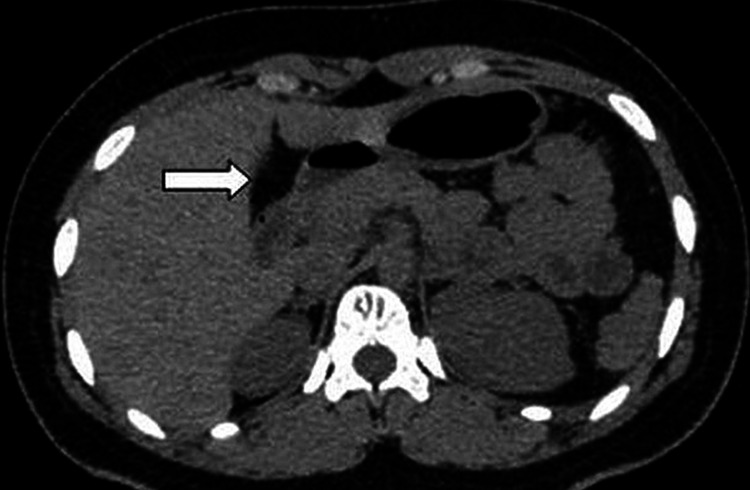
Axial non-contrast CT showing fat in the gallbladder fossa (arrow) with non-visualization of the gallbladder. CT - Computed Tomography.

The patient was reviewed again with overnight fasting for the purpose of visualization of the gallbladder. The gallbladder still was not visualized on ultrasound in the gallbladder fossa. Computed tomography (CT) was done to rule out possible calculi or chronic cholecystitis. The gallbladder was not visualized in the gallbladder fossa, and there were no radio-dense calculi or inflammation in pericholecystic areas (Figures 2, 3). The gold standard test to confirm chronic cholecystitis is the hepatobiliary iminodiacetic acid (HIDA) scan. However, the patient deferred the HIDA scan due to the cost.

 

**Figure 4:**
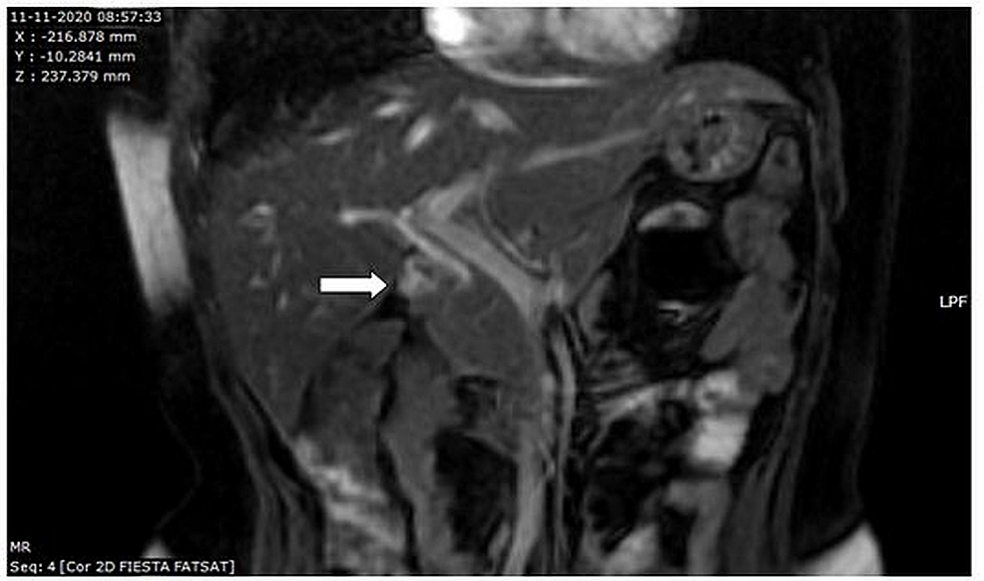
Coronal 2D FIESTA sequence of MRCP showing non-visualization of the gallbladder in the gallbladder fossa with normal common hepatic and common bile ducts. 2D FIESTA - Two-Dimensional Fast Imaging Employing Steady-state Acquisition; MRCP - Magnetic Resonance Cholangiopancreatography

MRCP was subsequently performed since the suspicion of gallbladder agenesis was put forth on CT. MRCP was done after overnight fasting. Routine MRCP sequences were performed with GE SIGNA 1.5 Tesla MRI. The gallbladder was not visualized in MRCP (Figure 4).

**Figure 5:**
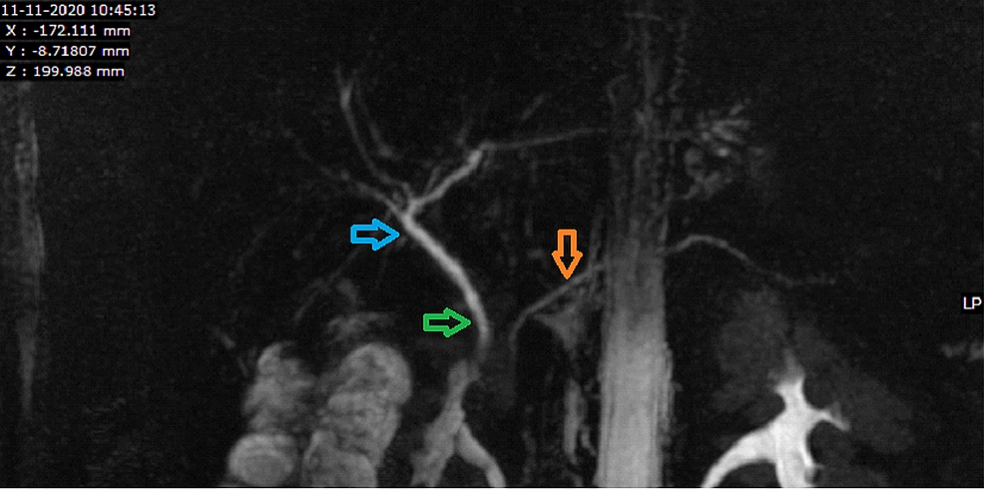
3D MRCP ASSET image showing a normal biliary tree with gallbladder absent. The common bile duct (green arrow), common hepatic duct (blue arrow), and pancreatic duct (orange arrow) appear normal in caliber and course. 3D MRCP ASSET - Three Dimensional array spatial sensitivity encoding technique.

The common bile duct, common hepatic duct, and rest of the biliary structures appeared normal (Figure 5).

The rest of the biliary tree was not dilated. No evidence of choledocholithiasis was observed.

The pancreatic parenchyma showed normal signal intensities with a normal-appearing pancreatic duct. No evidence of inflammation or fluid was observed in the peritoneal cavity. Review by the gastroenterologist concluded as the patient was asymptomatic after antispasmodics, and no other investigations were opted for. The patient was discharged on proton pump inhibitors and antispasmodics. Possible future symptoms were discussed, and a yearly follow-up was advised.

## Discussion

The liver and gallbladder develop in the fourth week of intrauterine life [6]. The gallbladder originates from the cystic duct as a ventral outgrowth from the caudal region of the foregut. The vacuolation of hyperplastic epithelium starts at about the seventh week, during which the gallbladder and cystic duct develop a lumen [7]. Though the exact pathogenesis is unknown, gallbladder agenesis is thought to be due to the failure of the cystic bud to develop further or to the failure of vacuolation [6]. Furthermore, anomalies at any stage of this process can result in gallbladder agenesis.

Occasionally, the gallbladder diverticulum formed from the hepatic diverticulum can inappropriately migrate, forming an ectopic gallbladder. The ectopic gallbladder is often seen in locations such as the posteroinferior surface of the liver and can also be retrohepatic, retropancreatic, or retroduodenal; sometimes, it can even be seen on the left side.

Gallbladder agenesis can be associated with several genitourinary, gastrointestinal, or cardiovascular malformations, including Klippel-Feil syndrome, cerebrotendinous xanthomatosis, Trisomy 18, malrotation of the gut, and heterotaxy syndromes [5-7].

Gallbladder agenesis is a rare congenital anomaly in which the majority of affected people are asymptomatic. Initial clinical suspicion is usually cholecystitis or biliary colic. When symptomatic, patients can present with signs of biliary colic, cholecystitis, or sometimes jaundice. Though there is no clear reason why some patients with gallbladder agenesis are symptomatic while others are not, a mechanism has been proposed whereby pain in gallbladder agenesis is due to biliary dyskinesia, Sphincter of Oddi dysfunction, or choledocholithiasis [2,3,8,9].

Several case reports published had a similar clinical presentation of upper abdominal pain resembling biliary colic, along with normal or elevated laboratory workup and unclear radiological signs [1-5,7,8-10]. Other common presentations include nausea and/or vomiting (66%), fatty food intolerance (37.5%), dyspepsia, bloating, and occasional jaundice [7].

 When a patient presents with symptoms of biliary colic or upper abdominal pain, the first investigation of choice would be an abdominal ultrasound. In order to allow the gall bladder to distend and to reduce the amount of gas present in the bowel and assist in distinguishing bowel loops from an absent gallbladder, the ultrasound should be performed after fasting [5]. The “WES” triad (wall-echo-shadow sign - demonstration of gallbladder Wall, Echo of stone, and acoustic Shadow) has been proposed to differentiate between a contracted gallbladder with gallstones and bowel loops [5,7].

A non-contrast CT scan of the abdomen shows an empty gallbladder fossa, with no radio-dense calculi seen in the biliary system (Figures 2, 3).

Thus, the findings of abdominal ultrasound can be misleading, and MRCP is indicated to confirm a diagnosis of gallbladder agenesis (Figures 4, 5) [1,4,5].

 MRCP, being a non-invasive procedure, is the investigation of choice for diagnosing this condition. MRCP does not require contrast administration, and the rest of the biliary tree can also be effectively assessed by this technique. The presence of an ectopic gallbladder can also be identified [4]. Other modalities, such as endoscopic retrograde cholangiopancreatography (ERCP) or endoscopic ultrasound, can be used for diagnosis but are invasive procedures and usually provide limited information on the presence of ectopic gallbladders.

After choledocholithiasis is excluded and the diagnosis of gallbladder agenesis is confirmed, conservative management with smooth muscle relaxants or biliary sphincterotomy for refractory cases can be provided [3].

## Conclusions

In summary, this case report of a young woman presenting with biliary colic symptoms mimicking chronic cholecystitis and found to have gallbladder agenesis serves as a reminder of a rare but convincing mimicry of cholecystitis, both clinically and radiographically. Though gallbladder agenesis is rare, clinical knowledge about the condition with suitable preoperative investigations provides reliable preoperative diagnosis and, therefore, helps in reducing morbidities due to operative interventions. Thus, combined clinical and radiological knowledge helps to provide better patient care.

## References

[1] Elzubeir N, Nguyen K, Nazim M (2020). Acute cholecystitis-like presentation in an adult patient with gallbladder agenesis: case report and literature review. Case Rep Surg.

[2] Tagliaferri E, Bergmann H, Hammans S, Shiraz A, Stüber E, Seidlmayer C (2016). Agenesis of the gallbladder: role of clinical suspicion and magnetic resonance to avoid unnecessary surgery. Case Rep Gastroenterol.

[3] Moon AM, Howe JH, McGinty KA, Gerber DA (2018). Gallbladder agenesis mimicking cholelithiasis in an adult. Radiol Case Rep.

[4] Pipia I, Kenchadze G, Demetrashvili Z, Nemsadze G, Jamburia L, Zamtaradze T, Abiatari I (2018). Gallbladder agenesis: a case report and review of the literature. Int J Surg Case Rep.

[5] Tsalikidis C, Gaitanidis A, Kavazis C (2020). A case of symptomatic gallbladder agenesis with chronic abdominal symptoms. Folia Med (Plovdiv).

[6] Fiaschetti V, Calabrese G, Viarani S, Bazzocchi G, Simonetti G (2009). Gallbladder agenesis and cystic duct absence in an adult patient diagnosed by magnetic resonance cholangiography: report of a case and review of the literature. Case Rep Med.

[7] Bianco G, Frongillo F, Agnes S, Nure E, Silvestrini N (2018). Gallbladder agenesis: a case report and brief review. Ann Hepatobiliary Pancreat Surg.

[8] Latimer EO, Mendez FL Jr, Hage WJ (1947). Congenital absence of gallbladder: report of three cases. Ann Surg.

[9] Malde S (2010). Gallbladder agenesis diagnosed intra-operatively: a case report. J Med Case Rep.

[10] Balakrishnan S, Singhal T, Grandy-Smith S, El-Hasani S (2006). Agenesis of the gallbladder: lessons to learn. JSLS.

